# Association of Environmental Cadmium Exposure with Pediatric Dental Caries

**DOI:** 10.1289/ehp.10947

**Published:** 2008-02-06

**Authors:** Manish Arora, Jennifer Weuve, Joel Schwartz, Robert O. Wright

**Affiliations:** 1 Environmental and Occupational Medicine and Epidemiology, Department of Environmental Health, Harvard School of Public Health, Boston, Massachusetts, USA; 2 Population Oral Health, Faculty of Dentistry, Westmead Centre for Oral Health, University of Sydney, Sydney, New South Wales, Australia; 3 Channing Laboratory, Department of Medicine, Brigham and Women’s Hospital, Harvard Medical School, Boston, Massachusetts, USA

**Keywords:** children, dental caries, environmental tobacco smoke, NHANES III, urine cadmium

## Abstract

**Background:**

Although animal experiments have shown that cadmium exposure results in severe dental caries, limited epidemiologic data are available on this issue.

**Objectives:**

We aimed to examine the relationship between environmental cadmium exposure and dental caries in children 6–12 years of age.

**Methods:**

We analyzed cross-sectional data, including urine cadmium concentrations and counts of decayed or filled tooth surfaces, from the Third National Health and Nutrition Examination Survey. We used logistic and zero-inflated negative binomial (ZINB) regression to estimate the association between urine cadmium concentrations and caries experience, adjusting these analyses for potential confounders including environmental tobacco smoke (ETS).

**Results:**

Urine cadmium concentrations ranged from 0.01 to 3.38 ng/mL. Approximately 56% of children had experienced caries in their deciduous teeth, and almost 30% had been affected by caries in their permanent dentition. An interquartile range (IQR) increase in creatinine-corrected cadmium concentrations (0.21 μg/g creatinine) corresponded to a 16% increase in the odds of having experienced caries in deciduous teeth [prevalence odds ratio (OR) = 1.16; 95% confidence interval (CI), 0.96–1.40]. This association was statistically significant in children with low ETS exposure (prevalence OR = 1.30; 95% CI, 1.01–1.67). The results from the ZINB regression indicated that, among children with any caries history in their deciduous teeth, an IQR increase in cadmium was associated with 17% increase in the number of decayed or filled surfaces. We observed no association between cadmium and caries experience in permanent teeth.

**Conclusions:**

Environmental cadmium exposure may be associated with increased risk of dental caries in deciduous teeth of children.

Dental caries is the most common chronic childhood disease in the United States (U.S. Department of Health and Human Services 2000). The prevalence of dental caries exceeds 50% in 5- to 9-year-old U.S. children and increases to 78% in those 17 years of age, making this disease more common than asthma and hay fever (U.S. Department of Health and Human Services 2000). Dental caries has been associated with numerous adverse effects on children’s health including pain, restricted dietary intake, impaired growth, and reduced body weight [reviewed by Shieham (2006)]. Children living below the poverty line have more severe dental caries, and many remain untreated because of low dental health insurance coverage (U.S. Department of Health and Human Services 2000). An increasing body of evidence supports the role of environmental factors in the etiology of dental caries. Exposure to lead and environmental tobacco smoke (ETS), which has high concentrations of cadmium, has been linked with an increased risk of dental caries in children (Aligne et al. 2003; Gemmel et al. 2002; Moss et al. 1999). These studies consistently reported positive associations between environmental exposures and caries in deciduous teeth (baby teeth) but not in permanent teeth (Aligne et al. 2003; Gemmel et al. 2002; Youravong et al. 2006), indicating that children’s deciduous dentition may be particularly susceptible to environmental toxicants.

An estimated 2.3% of Americans have elevated urine cadmium concentrations, a bio-marker of cumulative cadmium exposure (Paschal et al. 2000). ETS, a known risk factor for dental caries in children, accounts for approximately 20% of urine cadmium levels in U.S. children (Mannino et al. 2002). Other sources of cadmium include emissions from mining, smelting, fuel combustion, phosphate fertilizer use, sewage sludge application, disposal of metal wastes, and industrial uses of cadmium in manufacturing of batteries, pigments, stabilizers, and alloys [Agency for Toxic Substances and Disease Registry (ATSDR) 1999]. Cadmium is present in trace amounts in certain foods such as leafy vegetables, potatoes, grains and seeds, liver and kidney, and crustaceans and mollusks (Satarug et al. 2003). Once in the body, cadmium accumulates in the kidney, liver, and bone and is excreted very slowly (ATSDR 1999).

Exposure to cadmium is associated with numerous systemic health effects including renal dysfunction, skeletal disorders, and cardiovascular disease (Jarup 2003). Furthermore, the International Agency for Research on Cancer (IARC) has classified cadmium as a Group I human carcinogen (IARC 1993). The evidence of an association between cadmium and dental caries arises from animal experiments. Shearer et al. (1980a) showed that exposure to cadmium in rats during the neonatal period resulted in the development of severe dental caries, and this caries-promoting effect of cadmium was not negated by the addition of fluoride to drinking water. Administration of cadmium also disrupted salivary gland function in rats (Abdollahi et al. 2000; Chiarenza et al. 1989).

We examined the association of environmental cadmium exposure with pediatric dental caries using data from the Third National Health and Nutrition Examination Survey (NHANES III), a nationally representative survey conducted from 1988 to 1994 in the United States. We placed particular emphasis on differentiating the association of cadmium exposure and dental caries from the effects of ETS, which may confound the relationship between cadmium and dental caries.

## Methods

### Study subjects

The NHANES III included personal household interviews and health examinations of approximately 30,000 civilian noninstitutionalized persons ≥ 2 months of age [National Center for Health Statistics (NCHS) 2006]. Data were collected on a large number of variables including demographics, education, income, diet, presence of smokers in household, and utilization of dental services. The physical examination included recording of dental caries and fillings in deciduous and permanent teeth, and collection of blood and urine samples for laboratory analyses. Participants gave written informed consent before joining the survey, and parents provided consent on behalf of children. For the present study, we included children 6–12 years of age who had undergone a dental examination and had laboratory measurements of cadmium and cotinine (a biomarker of exposure to tobacco smoke). In the NHANES III, urine cadmium was measured only for children ≥ 6 years of age, and we excluded children > 12 years of age because deciduous teeth have generally shed beyond this age (Berkovitz 1992). Of the children in this age range, 3,006 children had urine cadmium and serum cotinine measurements. We excluded 26 children with missing urine creatinine measurements and another 24 children with high serum cotinine levels. Of these children, 2,315 and 2,886 had dental caries and fillings recorded in deciduous and permanent teeth respectively.

### Urine cadmium and creatinine measurements

Details of the urine collection and cadmium analysis have been reported previously (Paschal et al. 2000). Briefly, during the physical examination a 10-mL spot sample of urine was collected from survey participants. Cadmium was quantified by Zeeman effect graphite furnace atomic absorption spectrophotometry, using the Centers for Disease Control and Prevention’s modification of the method of Pruszkowska et al. (1983). The detection limit was approximately 0.01 ng/mL (NCHS 2006). Each specimen was analyzed in duplicate, and mean measurements were reported. All specimens with cadmium concentrations < 5 μg/L were reanalyzed for confirmation (Paschal et al. 2000). We calculated creatinine-corrected urine cadmium values as 100 times the ratio of urine cadmium to urine creatinine (Paschal et al. 2000).

### Dental examination

Details of the dental component of the NHANES III have been reported previously (Drury et al. 1996; Selwitz et al. 1996). Briefly, trained and calibrated dentists performed a visual–tactile examination of the oral cavity. Teeth were dried before the dental caries assessment. Quality control was maintained by various procedures including training and calibration of staff, the use of a standard examiner, and ongoing monitoring of interexaminer reliability and consistency with the standard examiner (Drury et al. 1996).

In evaluating the deciduous dentition, we used the sum of decayed and filled deciduous tooth surfaces (dfs). For children with at least one permanent tooth, we also included the sum of their decayed, filled, and missing permanent tooth surfaces (DMFS) to evaluate caries in permanent teeth. We undertook separate analyses for caries scores in deciduous and permanent teeth.

### Assessment of exposure to ETS

Because ETS is a major source of inhaled cadmium in children (Mannino et al. 2002) and is also associated with caries risk (Aligne et al. 2003), it is an important potential confounder of any observed association between cadmium exposure and caries experience. The NHANES III assessed children’s exposure to ETS in several ways, and we incorporated two of these assessments into our study. The NHANES III measured serum cotinine, a biomarker of ETS exposure, using isotope dilution liquid chromatography with tandem mass spectrometry (Gunter et al. 1996), with a limit of detection of 0.05 ng/mL (NCHS 2006). We also used a variable that recorded the number of persons who smoked cigarettes at the child’s home. We considered children with no cigarette smokers at home and with serum cotinine levels < 0.2 ng/mL as being exposed to low levels of ETS. This serum cotinine cutoff level has been used in previous investigations of the relation of ETS to pediatric dental caries in the NHANES III (Aligne et al. 2003). We excluded 24 participants with serum cotinine levels > 10 ng/mL, who may have been early active smokers.

### Statistical analysis

We examined summary statistics and scatterplots for urine cadmium, serum cotinine, dfs, and DMFS scores to identify outliers. We also studied the distribution of creatinine-corrected urine cadmium concentrations and dfs and DMFS scores within key subject characteristics. Subsequently, we used multivariable-adjusted logistic regression to estimate the prevalence odds ratio (OR) of having some caries experience per microgram per gram creatinine-corrected urine cadmium concentration. Again, we examined caries experience in deciduous teeth (dfs ≥ 1) and in permanent teeth (DMFS ≥ 1) separately. The principal models incorporated variables previously associated with dental caries including age (years), sex, education of head of household (< high school, high school, or > high school), household income (≥ 100% of federal poverty line vs. < 100% of federal poverty line), number of smokers in household (none vs. ≥ 1), ethnicity (non-Hispanic white, non-Hispanic black, Mexican American, and other), blood lead level (micrograms per deciliter), serum cotinine (nanograms per milliliter), and sucrose intake (grams per day). We considered the influence of vegetable intake in our analyses because certain vegetables may contain cadmium (Satarug et al. 2003), and vegetable intake has been associated with a reduced risk of caries in some children (Dye et al. 2004). We also tested the impact of including time since last visit to dentist, region of residence, and residence in metropolitan area. These variables were not significant predictors of dental caries and did not change the estimated association between urine cadmium and dental caries scores, and therefore we excluded these variables from the final model. Furthermore, we considered the possibility that consumption of fluoridated water might be associated with cadmium exposure and thereby could account for some or all of the association we observed. To address this possibility in the absence of water fluoridation data, we conducted a sensitivity analysis, based on the work of Bross (1966) and Walker (1991) [see Supplemental Material for details (online at http://www.ehponline.org/members/2008/10947/suppl.pdf)].

A limitation of using logistic regression to evaluate the relationship of cadmium to caries experience is that it does not consider the range of caries experience beyond “any” carious, filled, or missing surfaces. Zero-inflated Poisson (ZIP) and zero-inflated negative binomial (ZINB) regression are variants of Poisson regression that offer the advantage of modeling dental caries scores on a continuum instead of the dichotomized outcome used in logistic regression, and thereby they assess the association of exposure variables with disease severity, not just the presence or absence of disease (Lambert 1992; Slymen et al. 2006). ZIP and ZINB regression are preferable to Poisson regression in modeling dental caries data because of the large proportion of participants with a zero dfs/DMFS score. The ZIP and ZINB regression models estimate both the relative odds of being caries free (dfs = 0, DMFS = 0) and the relative caries index among those who are not caries free. Böhning et al. (1999) and Lewsey and Thomson (2004) provide a detailed discussion of the utility of ZIP and ZINB regression in modeling dental caries data. In our analyses, we found that the ZINB regression model fit the data better than the ZIP regression model (data not shown), and therefore we only report the results of the ZINB regression analysis. In both logistic and ZINB regression analyses, we modeled creatinine-corrected urine cadmium as a continuous term, and then used the resulting regression parameter to compute the association estimates of an interquartile range (IQR) increase in creatinine-corrected urine cadmium (0.21 μg/g creatinine).

We also considered that differences between participants in the number of primary and permanent teeth present in their oral cavity may influence the observed association between cadmium and dental caries, particularly in the ZINB regression analysis. We therefore undertook additional analyses to account for any such effect. For deciduous teeth, we restricted our sample to children having at least two of possible eight molars and used caries scores from molars only in the regression models. In addition, we also included in the ZINB model an “offset” term for the log of the number of deciduous surfaces at risk for caries for each child. For both these approaches, we compared changes in association between cadmium and caries scores from the original analysis, which included all deciduous teeth. We also undertook similar analyses for permanent teeth where we restricted our sample to children with first permanent molars.

Finally, we were concerned that our findings could be residually confounded by exposure to ETS that was not accounted for by serum cotinine or reports of smokers in the home. Therefore, we conducted parallel analyses restricted to children who had very low exposure to ETS (serum cotinine levels < 0.2 ng/mL and no reported cigarette smokers at home).

For data analyses, we used SAS version 9.1 (SAS Institute Inc., Cary, NC, USA) and Intercooled STATA 10.0 (StataCorp., College Station, TX, USA), and all analyses accounted for the complex multistage sampling design of the NHANES III.

## Results

In our study sample, the unadjusted urine cadmium levels ranged from 0.01 to 3.38 ng/mL with a median of 0.13 ng/mL. Approximately 56% of the children had experienced caries in their deciduous teeth (dfs ≥ 1), and almost 30% had been affected by caries in their permanent dentition (DMFS ≥ 1). The geometric mean creatinine-corrected urine cadmium concentrations within key subject characteristics are given in Table 1. We observed higher cadmium concentrations in children belonging to families where the head of the household had not attained education beyond high school, income was below the federal poverty line, and there was more than one cigarette smoker at home. Non-Hispanic black and Mexican-American children also had higher cadmium concentrations than non-Hispanic white children. We also observed positive associations of urine cadmium with blood lead and serum cotinine.

Data have been previously published, from the NHANES III, describing the distribution of childhood dental caries between different sociodemographic strata (Vargas et al. 1998) and other variables pertaining to our study, including exposure to ETS, sucrose intake, and blood lead levels (Moss et al. 1999). We observed that in the deciduous dentition, a history of caries was more common among children from families where the household head had lower education levels and among children from households with income below the federal poverty line. Similarly, a history of caries was more prevalent among Mexican-American children and children of “other” ethnic groups than among non-Hispanic white children. Higher serum cotinine and blood lead levels were also associated with having experienced some caries in deciduous teeth. These differences were statistically significant (*p* < 0.05). We observed similar differences in caries experience in the permanent dentition in relation to education of household head, ethnicity, and age of the child. Furthermore, females were more likely to have experienced caries in their permanent dentition than males. Children whose creatinine-corrected urine cadmium concentrations were above the median were more likely to have experienced caries. These differences were not significant for permanent teeth and were only marginally statistically significant for deciduous teeth (*p* < 0.08).

After adjusting for numerous potential confounding factors, we observed that creatinine-corrected urine cadmium levels were associated with greater prevalence of caries history and greater severity of caries experience in deciduous teeth. The results of our logistic regression analysis (Table 2) indicated that an IQR increment in cadmium concentrations (0.21 μg/g creatinine) corresponded to a 16% increase in the odds of having experienced caries in our study sample [OR = 1.16; 95% confidence interval (CI), 0.96–1.40]. In children with low ETS exposure, this amounted to an increase in odds of 30% (OR = 1.30; 95% CI, 1.01–1.67). Lower education level of household head, age, and blood lead and serum cotinine levels were also associated with a higher risk of having experienced caries.

Results from the ZINB regression analysis (Table 2) indicated that urine cadmium levels were not only associated with decreased odds of remaining caries free (a confirmation of the logistic regression results in multiplicative inverse) but also with a greater number of carious or filled surfaces. Similar to the logistic regression results, this association was statistically significant in children classified as having low exposure to ETS. In children exposed to low ETS, an IQR increase in creatinine-corrected urine cadmium concentrations corresponded to a 27% reduction in the odds of being caries free (OR = 0.73; 95% CI, 0.54–0.98) and a 17% increase in the number of deciduous carious or filled surfaces (relative count = 1.17; 95% CI, 1.02–1.33). Furthermore, analyses restricted to molars and those including an “offset” term resulted in only minor changes (< 10%) to the parameter estimates for the cadmium variable. Similar to analyses including all teeth, the association between cadmium and dental caries in deciduous teeth was statistically significant only in children with low ETS exposure. We conducted similar logistic and ZINB regression analyses to measure the association of cadmium exposure with caries experience in permanent teeth and observed no remarkable association (data not shown).

Finally, we also evaluated potential confounding by consumption of fluoridated water, using a sensitivity analysis, and found that only under extreme and sometimes implausible conditions could our findings be substantially confounded by this variable [see Supplemental Material for details (online at http://www.ehponline.org/members/2008/10947/suppl.pdf)].

## Discussion

Using data from the nationally representative NHANES III, we observed a positive, although statistically nonsignificant, association between environmental cadmium exposure and caries experience in deciduous teeth of U.S. children 6–12 years of age. When we restricted this analysis to children with low ETS exposure and adjusted for multiple potential confounders, the association between creatinine-corrected urine cadmium and caries experience was statistically significant. Using logistic regression analyses, we observed that cadmium exposure was associated with increased odds of having experienced any caries. The results from our ZINB regression analysis, in addition to confirming the results of the logistic regression, also showed that cadmium exposure was associated with increased severity of caries experience. These results remained essentially unchanged when we restricted our analysis to deciduous molars and adjusted for the number of tooth surfaces at risk of caries.

To the best of our knowledge, this is among the first epidemiologic studies reporting an association between cadmium exposure and caries in the deciduous teeth of children. Our findings are consistent with previously reported data in humans and a number of animal experiments. Curzon and Crocker (1978) reported a positive correlation between cadmium levels in enamel of human premolar teeth and caries scores in permanent teeth. Kobylanska et al. (1969) observed that industrial workers exposed to cadmium fumes developed a yellowish discoloration of teeth and had high levels of caries. In animal experiments, administration of cadmium to young rats for 150 days resulted in the development of higher levels of caries compared with controls (Ginn and Volker 1944). In shorter-duration experiments, Leicester (1946) reported increased rates of caries progression in rats that were given cadmium in drinking water. Later studies by Shearer et al. (1980a, 1980b) showed that cadmium exposure in rats during the period of tooth development caused severe caries and also partially negated the cariostatic influence of fluoride. Cadmium administration after tooth development did not increase caries (Shearer et al. 1980b). In our study, environmental cadmium exposure was associated with caries scores in deciduous teeth but not in the permanent teeth. Furthermore, some epidemiologic investigations of environmental lead exposure have also observed significant associations with caries only in deciduous teeth (Gemmel et al. 2002; Youravong et al. 2006), suggesting that deciduous teeth may be more susceptible than permanent teeth to environmental toxicants. However, we cannot rule out that early-life cadmium exposure also affects permanent dentition, and long-term prospective studies with repeated cadmium exposure measurements are needed to address this concern.

The caries-promoting effect of cadmium may be linked to disruption of salivary gland function. Subcutaneous administration of cadmium to rats produced histologic signs of tubular and acinar damage in salivary glands. Furthermore, cadmium exposure depressed salivary secretions and reduced the concentration of the major salivary digestive enzyme, amylase, in parotid gland saliva (Chiarenza et al. 1989). It has been suggested that these effects of cadmium may be attributable to the inhibition of acetylcholine release and the disruption of parasympathetic impulses, which play a major role in regulating salivary secretions (Cooper and Manalis 1983). Competitive inhibition of calcium channels by cadmium (Abdollahi et al. 2000; Blazka and Shaikh 1991) and induction of oxidative stress in salivary glands (Abdollahi et al. 2003) have also been reported.

We found that the association between cadmium and dental caries was statistically significant in children with low ETS exposure, as defined by serum cotinine concentrations < 0.2 ng/mL and absence of cigarette smokers at the child’s home. ETS contains several risk factors for dental caries, including 250 chemicals known to be toxic or carcinogenic (National Toxicology Program 2005), and has been associated with an increased risk of dental caries in children surveyed in the NHANES III (Aligne et al. 2003) and in other populations (Shenkin et al. 2004). It is therefore possible that competing risk factors for dental caries due to ETS exposure obscure the association of cadmium and dental caries in children exposed to ETS. This complication is compounded by the limited sensitivity of the dfs score as a measure of disease severity (Kingman and Selwitz 1997). For example, the dfs score does not distinguish between carious lesions of different size.

Other findings of our study, including the positive associations of dental caries scores with blood lead and serum cotinine, confirm the results of earlier investigations using data from NHANES III and also studies conducted in other populations (Aligne et al. 2003; Gemmel et al. 2002; Moss et al. 1999; Youravong et al. 2006). The variations in dental caries scores across different strata of income, ethnicity, and education that we observed have been reported previously (Vargas et al. 1998).

Our study is strengthened by the use of well-established biomarkers of cadmium and ETS exposure, as well as detailed measures of caries experience and a number of important covariates. Although the cross-sectional nature of our study does not establish the temporal order of cadmium exposure and caries development, it seems unlikely that reverse causation is at play such that caries experience influences the metabolism of cadmium. The proportion of cadmium in teeth, relative to cadmium in other tissues such as kidney, liver, or bone, is small. Any release of cadmium from caries-affected teeth would not be expected to significantly increase urinary cadmium concentrations. Moreover, urine cadmium concentrations reflect exposures over the past decade or longer (Lauwerys et al. 1994), making it possible that the observed cadmium exposures preceded some or all of the caries experience for these children.

Although we have adjusted our analyses for numerous potential confounders, it is possible that our results remain confounded either by mismeasured or unmeasured covariates. Because consumption of fluoridated water is believed to protect against caries, and because its relation to cadmium exposures is not known, we were concerned that this factor may have partially or wholly confounded our results. Based on results from our sensitivity analyses [see Supplemental Material for details (online at http://www.ehponline.org/members/2008/10947/suppl.pdf)], it appears unlikely that differences in water fluoridation could explain the observed association between cadmium exposure and caries experience. Although the biological evidence supports the role of cadmium in disruption of salivary gland function, the NHANES III does not include any direct measures of salivary gland function, which limits further investigation, using NHANES III data, into the mechanisms behind the observed association between cadmium and dental caries. Furthermore, the NHANES III collects urine cadmium measurements only from children ≥ 6 years of age, not allowing investigation of younger children.

Overall, our study provides evidence that cadmium may be associated with an increased risk of dental caries in deciduous teeth of children. Because of numerous sources of exposure and widespread distribution of this toxicant, it is increasingly important to understand the systemic and oral health effects of cadmium, particularly in highly susceptible populations such as children. Prospective epidemiologic studies are needed to confirm these findings and to understand the mechanisms behind the observed association between cadmium and dental caries.

## Figures and Tables

**Table 1:** Distribution of creatinine-corrected urine cadmium concentrations by participant characteristics.

Characteristic	No. of children^a^ (unweighted)	Geometric mean urine cadmium (μg/g creatinine) (95% CI)	*p*-Value^b^
dfs			0.09
0	1,027	0.080 (0.074–0.087)	
≥ 1	1,288	0.088 (0.082–0.095)	
DMFS			0.61
0	1,976	0.086 (0.081–0.091)	
≥ 1	910	0.088 (0.081–0.096)	
Sex			0.004
Male	1,511	0.080 (0.075–0.086)	
Female	1,445	0.092 (0.086–0.099)	
Age (years)			0.65
6–7	811	0.083 (0.075–0.091)	
8–9	855	0.087 (0.080–0.096)	
10–12	1,290	0.087 (0.081–0.093)	
Education level of household head			< 0.0001
< High school	706	0.101 (0.091–0.111)	
High school	1,495	0.092 (0.086–0.098)	
> High school	755	0.064 (0.058–0.071)	
Race/ethnicity			< 0.0001
Non-Hispanic white	759	0.067 (0.061–0.074)	
Non-Hispanic black	1,006	0.096 (0.088–0.104)	
Mexican American	1,061	0.092 (0.085–0.100)	
Other	130	0.085 (0.069–0.106)	
Region			0.08
Northeast	302	0.084 (0.072–0.098)	
Midwest	538	0.086 (0.077–0.096)	
South	1,258	0.081 (0.075–0.087)	
West	858	0.094 (0.087–0.103)	
Metro vs. nonmetro^c^			0.26
Metro	1,432	0.088 (0.082–0.095)	
Other	1,524	0.084 (0.078–0.090)	
Poverty status			< 0.0001
< Federal poverty line	1,149	0.105 (0.097–0.113)	
≥ Federal poverty line	1,584	0.072 (0.067–0.077)	
No. of persons smoking cigarettes at home			< 0.0001
≤ 1	1,824	0.079 (0.074–0.084)	
> 1	1,130	0.098 (0.091–0.106)	
Time since last visit to dentist (years)			0.99
≤ 1	2,415	0.086 (0.081–0.091)	
> 1	541	0.086 (0.076–0.096)	
Serum cotinine (ng/mL)			< 0.0001
< 0.08	741	0.072 (0.065–0.079)	
0.08–0.23	737	0.082 (0.074–0.091)	
0.23–0.79	739	0.090 (0.082–0.099)	
> 0.79	739	0.102 (0.093–0.113)	
Blood lead (μg/dL)			< 0.0001
<1.6	757	0.068 (0.062–0.075)	
1.6–2.6	723	0.076 (0.069–0.084)	
2.6–4.3	751	0.092 (0.084–0.102)	
> 4.3	719	0.114 (0.104–0.125)	
Sucrose intake (g/day)			0.01
< 27.3	713	0.095 (0.086–0.105)	
27.3–44.5	708	0.086 (0.078–0.095)	
44.5–68.6	711	0.075 (0.068–0.083)	
> 68.6	710	0.088 (0.079–0.097)	

a Number of subjects differ because of missing data.

b *p*-Values from *F*-test for difference in geometric mean cadmium concentrations between groups.

c Counties of metropolitan (metro) areas with population of ≥ 1 million or more were classified as metro areas.

**Table 2:** Multivariable-adjusted^a^ association between creatinine-corrected urinary cadmium^b^ and decayed or filled surfaces in deciduous teeth of children.

	All children (*n* = 2,041)^d^	Children with low ETS exposure^c^ (*n* = 870)^e^
Logistic regression		
Relative odds of having decayed or filled surfaces	1.16 (0.96–1.40)	1.30 (1.01–1.67)
ZINB regression		
Relative odds of having no decayed or filled surfaces	0.83 (0.63–1.08)	0.73 (0.54–0.98)
Relative number of decayed or filled surfaces^f^	1.04 (0.99–1.11)	1.17 (1.02–1.33)

a Models adjusted for age, sex, ethnicity, education level, poverty status, log-transformed blood lead, log-transformed serum cotinine, and dietary sucrose intake.

b Per interquartile increment in creatinine-corrected urine cadmium (0.21 μg/g creatinine).

c Children with serum cotinine levels < 0.2 ng/mL and no cigarette smoker at home.

d Six outliers excluded from analysis.

e Five outliers excluded from analysis.

f Values > 1 indicate more decayed or filled surfaces with greater exposure. For example, among children with low ETS exposure, an IQR increment in urine cadmium (0.21 μg/g creatinine) is associated with 17% more affected surfaces in children with any decayed or filled tooth surfaces.

## References

[b1-ehp0116-000821] Abdollahi M, Bahreini-Moghadam A, Emami B, Fooladian F, Zafari K (2003). Increasing intracellular cAMP and cGMP inhibits cadmium-induced oxidative stress in rat sub-mandibular saliva. Comp Biochem Physiol C Toxicol Pharmacol.

[b2-ehp0116-000821] Abdollahi M, Dehpour A, Kazemian P (2000). Alteration by cadmium of rat submandibular gland secretory function and the role of the l-arginine/nitric oxide pathway. Pharmacol Res.

[b3-ehp0116-000821] Aligne CA, Moss ME, Auinger P, Weitzman M (2003). Association of pediatric dental caries with passive smoking. JAMA.

[b4-ehp0116-000821] ATSDR (1999). Toxicological Profile for Cadmium.

[b5-ehp0116-000821] Berkovitz BKB (1992). A Color Atlas and Text or Oral Anatomy: Histology and Embryology.

[b6-ehp0116-000821] Blazka ME, Shaikh ZA (1991). Differences in cadmium and mercury uptakes by hepatocytes; role of calcium channels. Toxicol Appl Pharmacol.

[b7-ehp0116-000821] Böhning D, Dietz E, Schlattmann P, Mendonca L, Kirchner U (1999). The zero-inflated Poisson model and the decayed, missing and filled teeth index in dental epidemiology. J R Stat Soc.

[b8-ehp0116-000821] Bross ID (1966). Spurious effects from an extraneous variable. J Chronic Dis.

[b9-ehp0116-000821] Chiarenza A, Elverdin JC, Espinal E, Giglio MJ (1989). Effects of cadmium on the function and structure of the rat salivary glands. Arch Oral Biol.

[b10-ehp0116-000821] Cooper GP, Manalis RS (1983). Influence of heavy metals on synaptic transmission: a review. Neurotoxicology.

[b11-ehp0116-000821] Curzon ME, Crocker DC (1978). Relationships of trace elements in human tooth enamel to dental caries. Arch Oral Biol.

[b12-ehp0116-000821] Drury TF, Winn DM, Snowden CB, Kingman A, Kleinman DV, Lewis B (1996). An overview of the oral health component of the 1988–1991 National Health and Nutrition Examination Survey (NHANES III-Phase 1). J Dent Res.

[b13-ehp0116-000821] Dye BA, Shenkin JD, Ogden CL, Marshall TA, Levy SM, Kanellis MJ (2004). The relationship between healthful eating practices and dental caries in children aged 2–5 years in the United States, 1988–1994. JADA.

[b14-ehp0116-000821] Gemmel A, Tavares M, Alperin S, Soncini J, Daniel D, Dunn J (2002). Blood lead level and dental caries in school-age children. Environ Health Perspect.

[b15-ehp0116-000821] Ginn JJ, Volker JF (1944). Effect of cadmium and fluorine on rat dentition. Proc Soc Exptl Biol Med.

[b16-ehp0116-000821] Gunter EW, Lewis BL, Koncikowski SM (1996). Laboratory Methods Used for The Third National Health and Nutrition Examination Survey (NHANES III).

[b17-ehp0116-000821] IARC (International Agency for Research on Cancer) (1993). Beryllium, cadmium, mercury, and exposures in the glass manufacturing industry. Monogr Eval Carcinog Risks Hum.

[b18-ehp0116-000821] Jarup L (2003). Hazards of heavy metal contamination. Br Med Bull.

[b19-ehp0116-000821] Kingman A, Selwitz RH (1997). Proposed methods for improving the efficiency of the DMFS index in assessing initiation and progression of dental caries. Community Dent Oral Epidemiol.

[b20-ehp0116-000821] Kobylanska M (1969). Effect of cadmium on teeth and mucosa. Dent Abstr.

[b21-ehp0116-000821] Lambert D (1992). Zero-inflated Poisson regression, with an application to defects in manufacturing. Technometrics.

[b22-ehp0116-000821] Lauwerys RR, Bernard AM, Roels HA, Buchet JP (1994). Cadmium: exposure markers as predictors of nephrotoxic effects. Clin Chem.

[b23-ehp0116-000821] Leicester HM (1946). The effect of cadmium on the production of caries in the rat. J Dent Res.

[b24-ehp0116-000821] Lewsey JD, Thomson WM (2004). The utility of the zero-inflated Poisson and zero-inflated negative binomial models: a case study of cross-sectional and longitudinal DMF data examining the effect of socio-economic status. Community Dent Oral Epidemiol.

[b25-ehp0116-000821] Mannino DM, Albalak R, Jones R (2002). Tobacco smoke exposure and urinary cadmium levels in US children: data from NHANES III. Am J Respir Crit Care Med.

[b26-ehp0116-000821] Moss ME, Lanphear BP, Auinger P (1999). Association of dental caries and blood lead levels. JAMA.

[b27-ehp0116-000821] National Toxicology Program (2005). 11th Report on Carcinogens. Research Triangle Park, NC:National Toxicology Program, National Institute of Environmental Health Sciences. http://ntp.niehs.nih.gov/ntp/roc/eleventh/profiles/s176toba.pdf.

[b28-ehp0116-000821] NCHS (2006). NHANES III Laboratory Data File Documentation; The Third National Health and Nutrition Examination Survey (NHANES III), 1988–94.

[b29-ehp0116-000821] Paschal DC, Burt V, Caudill SP, Gunter EW, Pirkle JL, Sampson EJ (2000). Exposure of the U.S. population aged 6 years and older to cadmium: 1988–1994. Arch Environ Contam Toxicol.

[b30-ehp0116-000821] Pruszkowska E, Carnrick GR, Slavin W (1983). Direct determination of cadmium in urine with use of a stabilized temperature platform furnace and Zeeman background correction. Clin Chem.

[b31-ehp0116-000821] Satarug S, Baker JR, Urbenjapol S, Haswell-Elkins M, Reilly PE, Williams DJ (2003). A global perspective on cadmium pollution and toxicity in non-occupationally exposed population. Toxicol Lett.

[b32-ehp0116-000821] Selwitz RH, Winn DM, Kingman A, Zion GR (1996). The prevalence of dental sealants in the US population: findings from NHANES III, 1988–1991. J Dent Res.

[b33-ehp0116-000821] Shearer TR, Britton JL, DeSart DJ (1980a). Influence of post-developmental cadmium on caries and cariostasis by fluoride. Environ Health Perspect.

[b34-ehp0116-000821] Shearer TR, Britton JL, DeSart DJ, Johnson JR (1980b). Influence of cadmium on caries and the cariostatic properties of fluoride in rats. Arch Environ Health.

[b35-ehp0116-000821] Sheiham A (2006). Dental caries affects body weight, growth and quality of life in pre-school children. Br Dent J.

[b36-ehp0116-000821] Shenkin JD, Broffitt B, Levy SM, Warren JJ (2004). The association between environmental tobacco smoke and primary tooth caries. J Public Health Dent.

[b37-ehp0116-000821] Slymen DJ, Ayala GX, Arredondo EM, Elder JP (2006). A demonstration of modeling count data with an application to physical activity. Epidemiol Perspect Innov.

[b38-ehp0116-000821] U.S. Department of Health and Human Services (2000). Oral Health in America: A Report of the Surgeon General—Executive Summary.

[b39-ehp0116-000821] Vargas CM, Crall JJ, Schneider DA (1998). Sociodemographic distribution of pediatric dental caries: NHANES III, 1988–1994. J Am Dent Assoc.

[b40-ehp0116-000821] Walker AM (1991). Observation and Inference: An Introduction to The Methods of Epidemiology.

[b41-ehp0116-000821] Youravong N, Chongsuvivatwong V, Geater AF, Dahlen G, Teanpaisan R (2006). Lead associated caries development in children living in a lead contaminated area, Thailand. Sci Total Environ.

